# Laparoscopic Takedown of Failed Fundoplication with Recurrent Hiatal Hernia Repair and Conversion to Roux-en-Y Gastric Bypass

**DOI:** 10.1007/s11695-025-08119-6

**Published:** 2025-07-28

**Authors:** Hadar Nevo, Mohamad Hamoud, Sham-Eldin Mokary, Samih Zoabi, Nasser Sakran

**Affiliations:** 1 Holy Family Hospital Nazareth, Nazareth, Israel; 2 The Azrieli faculty of Medicine, Bar-Ilan University, Ramat Gan, Israel

**Keywords:** Revisional bariatric surgery, Roux-en-Y gastric bypass, Hiatal hernia, Gastroesophageal reflux disease, Obesity

## Introduction

Obesity is a well-established risk factor for gastroesophageal reflux disease (GERD) and continues to pose a significant public health challenge worldwide [1]. Nissen Fundoplication (NFP) is a widely accepted and effective surgical treatment for patients with GERD who fail to respond to maximal medical therapy [2]. However, in obese patients, hiatal hernia repairs with NFP are associated with higher recurrence rates [3]. Common mechanisms of failure include wrap herniation and intrathoracic migration, often necessitating revisional surgery [4].

Managing patients with failed fundoplication is particularly challenging due to altered anatomy and increased technical challenges. Laparoscopic Roux-en-Y gastric bypass (RYGB) has emerged as a safe and effective bariatric option, especially for patients with obesity or severe obesity and GERD. It has shown promising results and a decreasing complication rate as surgeons gain more experience [5].

This video demonstrates a complex revisional surgery involving recurrent hiatal hernia repair with conversion to RYGB in a patient with class II obesity.

## Methods and Procedures

A 53-year-old female with a history of Nissen fundoplication performed ten years earlier for GERD presented with recurrent heartburn, regurgitation, and class II obesity (BMI 38 kg/m^2^). A comprehensive preoperative evaluation included abdominal computed tomography, upper and lower gastrointestinal endoscopy, high-resolution esophageal manometry, and 24-h impedance-pH monitoring.

Diagnostic findings revealed slippage of the NFP wrap and a large recurrent hiatal hernia. No other abnormalities were noted. Given the symptomatic failure of the initial anti-reflux surgery and the patient’s obesity, a laparoscopic conversion from NFP to RYGB concurrent hiatal hernia repair was planned.

## Results

**Fig. 1:**
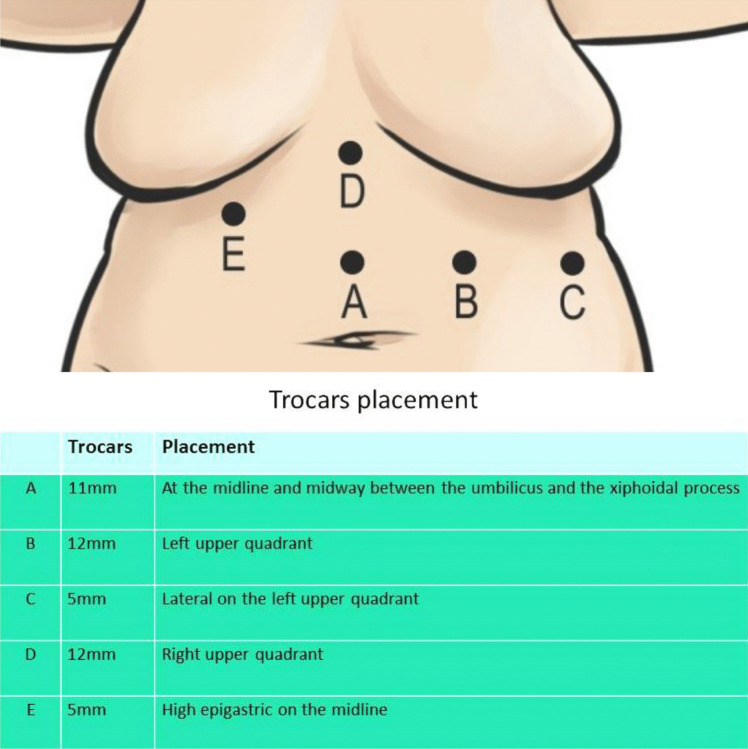
Trocars placement positions

The procedure was performed laparoscopically with the patient in the supine, split-leg position and in reverse Trendelenburg. A five-port technique is employed (Fig. 1). Adhesiolysis was first carried out to expose the right crus, which was separated from the caudate lobe. The left lobe of the liver was retracted to expose the hiatus. Dissection of the esophagus and stomach from the right and left crura was performed, followed by mediastinal dissection to achieve adequate esophageal mobilization. The previous Nissen wrap was taken down using a harmonic scalpel, preserving the vagus nerve and gastric blood supply. At least 3–4 cm of esophagus was mobilized intra-abdominally without tension.

A hiatal hernia repair was then completed. A gastric pouch was created around a 36 Fr gastric lavage tube, and a stapler was used to complete the pouch toward the angle of His. The ligament of Treitz was identified, and a jejunal loop was brought up antecolically, rotated, and anastomosed to the gastric pouch 100 cm distal to the ligament. A gastrojejunostomy was performed using a 45-mm linear stapler, ensuring correct alignment of the afferent and efferent limbs to avoid torsion. Stapler openings were closed with absorbable sutures. The Roux limb measured 100 cm in length.

A linear 60-mm stapler with a white cartridge was used to create a side-to-side jejunojejunostomy. The resulting enterotomy was lifted using three holding sutures (PDS II 4/0, Ethicon, USA) and closed longitudinally with a stapler. The remaining blind loop of the biliopancreatic limb was transected and removed using a similar linear stapler. A methylene blue leak test was negative. No intraoperative complications occurred.

Postoperatively, the patient was prescribed proton pump inhibitors for six months. She was discharged on postoperative day 3, remained asymptomatic, and had a BMI of 24.5 kg/m^2^ at six months. At 12 months, she achieved 89% excess weight loss with no recurrence of GERD.

## Conclusions

Bariatric surgery in patients with a history of anti-reflux procedures can be technically demanding. However, when performed in high-volume centers by experienced surgeons, it is a feasible and effective treatment option for obese patients with recurrent GERD.

## Supplementary Information

Below is the link to the electronic supplementary material.

Supplementary file1 (MP4 837107 KB)

## Data Availability

No datasets were generated or analysed during the current study.
